# Gender-Based Susceptibility to Mental Health Issues in Adolescents During the COVID-19 Pandemic: Descriptive Study

**DOI:** 10.2196/63284

**Published:** 2025-06-25

**Authors:** Young-Shin Lee, Minjeong Kim, Kim Moreno

**Affiliations:** 1 School of Nursing, San Diego State University, 5500 Campanile Dr., San Diego, CA, 92182, United States, 1 6195945385, 1 6195942765

**Keywords:** adolescent, anxiety, COVID-19, depression, post-traumatic stress disorder, mental health, coping strategies

## Abstract

**Background:**

Adolescence, the transitional phase between childhood and adulthood, is a stressful, fragile, and critical period. While the COVID-19 pandemic introduced numerous stressors affecting the mental health of all age groups, adolescents are particularly vulnerable. However, there is limited research focusing on the impact of COVID-19 on this population.

**Objective:**

This study aimed to explore the impact of COVID-19, coping strategies, depression, anxiety, and post-traumatic stress disorder (PTSD) in adolescents during the pandemic.

**Methods:**

This web-based cross-sectional survey study included 217 adolescents in Southern California, USA, between 2020 and 2021. Self-report measures include demographic questionnaires, COVID-19 impact, types of coping strategies used during COVID-19, depression, anxiety, and PTSD analyses. The *χ*^2^ tests were used for depression and anxiety, and ANOVA was used for PTSD analysis.

**Results:**

Female gender was identified as a risk factor for depression, anxiety, and PTSD. Approximately 24.2% (52/217) of participants had a family member or someone close who was infected with COVID-19 during the pandemic, which was a significant factor for both anxiety and PTSD (*P*<.05). More than half of all the participants (56.8%, 109/217) were Asian Americans, but there was no significant difference in depression, anxiety and PTSD among different racial or ethnic groups (*P*>.05). About a quarter of the participants reported experiencing depression (49/217, 25.5%) or anxiety (46/217, 24.0%). The mean (SD) score for PTSD was 8.08 (5.70). Social media and the internet were the most frequently used coping strategies, with 67.3% of participants using each.

**Conclusions:**

Considering our findings, prevention programs focusing on mental health, including routine screening, should be implemented at community level for adolescents. School programs fostering healthy social interactions and education on coping strategies should also be implemented for both families and adolescents.

## Introduction

### Pandemics and Mental Health of Adolescents

The COVID-19 pandemic has significantly affected everyone, particularly adolescents [1]. Adolescents, being in a crucial developmental stage, are particularly vulnerable to mental health issues. Their emotional regulation systems mature before those responsible for decision-making do, increasing their susceptibility to anxiety, aggression, and risky behaviors [2]. Socially, they rely more on peer interactions for development, with schools and community groups playing key roles in their socialization [3,4]. These interactions, vital for combating issues like low self-esteem and depression, were significantly disrupted by early COVID-19 public health recommendation including social distancing, limited interaction with peers, and school closures. Such disruptions could lead to long-term psychological and physiological impacts, highlighting the need to focus on adolescent mental health during the pandemic, given their vulnerability [4,5]. Therefore, it is important to understand the adolescent mental health status during COVID-19, given their susceptibility to mental health challenges.

The attention on adolescents’ health during COVID-19 pandemics is limited, especially in the United States, with most studies conducted in China, Australia, and Japan [3,6-10]. Studies on Chinese adolescents during the COVID-19 pandemic reported depression rates between 19.7% and 43.7%, anxiety rates between 24.9% and 37.4%, and a post-traumatic stress disorder (PTSD) rate of 14.4% [6-9]. In Japan, Isumi et al. [10] reported a 1.34-fold increase in child suicide rates in May 2020 compared to March 2020. Australian adolescents saw significant rises in depression and anxiety, alongside drops in life satisfaction during the pandemic [3].

The shift to online learning and reduced physical and social activity has marked a drastic change in adolescents’ lifestyles, contributing to increased screen time and more sedentary behaviors [11]. In the United States, there was a 50.6% rise in emergency department visits by adolescent girls for suicide attempts in February 2021 compared to 2019 [12]. Interestingly, US Hispanic adolescents with mental health issues before the COVID-19 pandemic experienced a significant decrease during the pandemic, possibly due to enhanced family interactions. However, the results of this study may not be generalized to the diverse US adolescents because of the large proportion of the Hispanic sample [1].

### Coping Strategies of Adolescents During COVID-19

Research on how adolescents adapted to restrictions on social and outdoor activities during the COVID-19 pandemic is scarce. One study highlighted that common coping strategies of young adults aged 18‐24 years included “just staying indoors” followed by “talking to people,” “maintaining a positive outlook,” and “trying to do some online school work” [13]. Another study found that children and adolescents engaged in spiritual/emotional activities; cognitive/social activities with family at home; exercise; and managing healthy sleep [14]. Despite the numerous stressors impacting the mental health of US adolescents during COVID-19, there was a gap in research specifically addressing the prevention and the mental health consequences for this at-risk group. Thus, examining the link between COVID-19 and mental health among US adolescents is crucial.

This study aims to investigate the association of COVID-19, coping mechanisms, and mental health challenges among adolescents in California during the pandemic. The specific aims are (1) to describe the prevalence of COVID-19 within the family or among someone close (through ties of kinship or affection); (2) to describe coping strategies used by adolescents; and (3) to identify whether demographic variables and being personally impacted by COVID-19 are related to depression, anxiety, and PTSD.

## Methods

### Ethical Considerations

This study was approved by the Institutional Review Board of the San Diego State University (HS-2020-0199). Both English and Spanish versions of the parent consent form were provided and signed by parents. All participants' identities were kept confidential. An electronic gift card of US $5 was sent to participants.

### Study Design and Setting

This study is a descriptive study using a cross-sectional design to identify the relationship between COVID-19 and mental health issues among adolescents in California. The web-based survey was conducted between October 2020 and February 2021, targeting adolescents in Southern California, United States. The inclusion criteria for this study were (1) boys and girls between 12 and 17 years old; (2) the ability to communicate verbally and/or in writing in English; (3) has access to an electronic device to complete the survey; and (4) both parent and participant provided consent for the survey study. The principal investigator assessed the participants’ eligibility. A convenience sampling method was used to recruit potential participants, including school nurses from Hispanic or Black backgrounds, parent associations, student science clubs, and by distributing flyers in shopping malls. A web-based self-report survey was developed using Qualtrics survey software (Qualtrics XM), and a survey link was sent to potential participants.

### Measures: Demographic Questionnaire

We included the gender (male and female), age (12‐14 years old and 15‐17 years old), and race/ethnicity (non-Hispanic White, Asian, and Hispanic).

#### COVID-19 Impact

The impact of COVID-19 was assessed using two questions with responses of yes/no: (1) family member/household infection with COVID-19, and (2) close contact death due to COVID-19. These were combined into a single variable if participants responded yes to either question. Regarding the coping strategies during COVID-19, participants indicated yes/no for various coping methods, including social media, internet use, video/computer games, sleep, eating, TV watching, phone calls, outdoor/indoor activities (walking, gardening, or shopping, etc.), indoor activities, and reading (books, newspapers, or magazines, etc.).

#### Mental Health Problems

The patient health questionnaire-4 (PHQ-4) was used to assess depression and anxiety using a 4-item, 4-point Likert scale. The depression subscale (PHQ-2) and anxiety subscale (GAD-2) each consist of 2 items, with scores ranging from 0 to 6. A score of 3 or greater on either subscale indicates a positive result for depression of generalized anxiety disorder (GAD). The PHQ-4 has established reliability and validity across diverse populations [15,16]. The impact of event scale-6 (IES-6) is a shorten version of the impact of event scale-revised (IES-R), comprising 6 items rated on a 5-point Likert scale to measure PTSD symptoms. The total scores range from 0 to 24, with higher scores indicating more severe post-traumatic stress reactions. The reliability and validity of the IES-6 have been established across various populations [17].

### Data Analysis

Descriptive statistics including the mean and SD were computed for all variables using the SPSS (version 27.0, IBM Corp). The *χ*^2^ tests were used to assess proportions of depression and anxiety by demographic variables and the COVID-19 impact. ANOVA analyses were employed to compare the mean PTSD score across demographic variables and the COVID-19 impact. The significance level for all statistical analyses was set at .05.

## Results

As of March 2021, a total of 217 adolescents participated in this study, including 108 males (50.2%), and 18% in the younger age group (12‐14 years old); Asian Americans were the largest ethnic group (109/217, 56.8%), followed by non-Hispanic White (52/217, 27.1%) and Hispanic (31/217, 16.1%; Table 1). Regarding the COVID-19 impact, approximately 24.2% (52/217) of the participants had a family member or someone close who had tested positive for COVID-19 or died from it.

**Table 1.** Demographic characteristics of participants and impact of COVID-19 (N=217).

Variable	n (%)
Gender	
Male	108 (50.2)
Female	107 (49.8)
Age	
Younger (12‐14 years old)	39 (18.0)
Older (15‐17 years old)	178 (72.0)
Race/Ethnicity	
Non-Hispanic White	52 (27.1)
Asian	109 (56.8)
Hispanic	31 (16.1)
COVID-19 impact	
Family or someone close tested positive for COVID-19	
Yes	52 (24.2)
No	163 (75.8)

Among the types of coping strategies, the use of social media and internet were the most common (146/217, 67.3%); followed by video or computer games, sleeping, eating, and TV watching (118/217, 54.4%); indoor activities (53/217, 24.4%); and reading books or magazines, which was the lowest (52/217, 24%; Table 2).

**Table 2.** Types of coping strategies during the COVID-19 pandemic (N=217).

Variable	n (%)^a^
Social media use	146 (67.3)
Internet use	146 (67.3)
Video or computer games	131 (60.4)
Sleeping	124 (57.1)
Eating	119 (54.8)
Watching TV	118 (54.4)
Talking with someone over the phone	94 (43.3)
Outdoor activities (walking, gardening, or shopping, etc.)	91 (41.9)
Indoor activities	53 (24.4)
Reading (books, newspapers, or magazines, etc.)	52 (24)

a Multiple responses were permitted.

Regarding the impact of COVID-19, the overall prevalence of depression was 25.5% (49/217) and that of anxiety was 24% (46/217); the overall mean (SD) score of PTSD was 8.08 (5.70; Table 3).

**Table 3.** Differences in depression, anxiety, and post-traumatic stress disorder (PTSD) by demographic characteristics and the COVD-19 impact (N=217).

Variable	Depression (PHQ-2^a^ score ≥3)			Anxiety (GAD-2^b^ score ≥3)			PTSD (IES-6^c^)		
	n (%)	χ² (*df*)	*P*	n (%)	χ² (*df*)	*P*	Mean (SD)	F test *(df)*	*P*
Gender									
Male	19 (17.6)	7.28 (1)	<.05	14 (13.0)	13.88 (1)	<.001	6.87 (5.10)	10.60 (1)	<.01
Female	36 (33.6)			37 (34.6)			9.34 (6.01)		
Age									
Younger (12‐14 years)	13 (31.0)	0.52 (1)	.47	11 (26.2)	0.06 (1)	.82	8.35 (5.78)	0.11 (1)	.74
Older (15‐17 years)	48 (25.5)			46 (24.5)			8.02 (5.70)		
Race/Ethnicity									
Non-Hispanic White	16 (30.8)	1.41 (2)	.5	14 (26.9)	0.61 (2)	.74	7.94 (5.41)	0.04 (2)	.96
Asian	27 (24.8)			26 (23.9)			8.12 (5.74)		
Hispanic	6 (19.4)			6 (19.4)			8.29 (5.74)		
COVID-19 impact: family or someone close was diagnosed with COVID-19									
Yes	19 (35.2)	3.68 (1)	.055	20 (37.0)	7.33 (1)	<.01	10.17 (6.31)	9.99 (1)	<.01
No	36 (22.1)			31 (19.0)			7.39 (5.33)		
Total	49 (25.5)			46 (24.0)			8.08 (5.70)		

a PHQ-2: depression subscale.

b GAD-2: anxiety subscale.

c IES-6: impact of event scale.

Female participants compared to male participants reported a significantly higher prevalence for depression (33.6% vs 17.6%, respectively) and anxiety (34.6% vs 13%, respectively; *P*<.05) and significantly higher mean (SD) scores for PTSD (9.34 [6.01] vs 6.87 [5.10], respectively; *P*<.01).

However, there were no significant differences in depression, anxiety, and PTSD (*P*>.05) among participants between age groups (12‐14 and 15‐17 years old) and racial/ethnic groups (Non-Hispanic White, Asian, and Hispanic). Participants who had a family member or someone close diagnosed with COVID-19 reported a significantly higher prevalence for anxiety compared to their counterparts (20/54, 37% vs 31/163, 19%, respectively; *P*<.01) and significantly higher mean scores for PTSD (10.17 vs 7.39, respectively; *P*<.01). The group experiencing COVID-19 did not show a significant difference in depression (35%) compared to those unaffected by COVID-19 (22%; *P*=.55).

## Discussion

### Findings and Comparison With Previous Works

The present study examined the association of the 2020‐2021 COVID-19 pandemic with adolescent depression, anxiety, and PTSD, as well as their coping strategies. Of the demographic variables, only female gender showed associations with all three mental health issues. These findings are consistent with findings from the United States and other countries [6,9,18]. While previous studies in China suggested older adolescents reported more mental health problems during the COVID-19 pandemic [8,9], our study found no significant age-related differences in mental health. The impact of COVID-19 on mental health among younger adolescents in the United States remains uncertain, warranting further studies. Notably, over half of the participants (56.8%) were Asian Americans, possibly influenced by heightened interest in the COVID-19 study due to prevalent anti-Asian discrimination and violence during the pandemic compared to other racial/ethnic groups.

Given that most studies about COVID-19 and adolescent mental health have been conducted in China [6,8,9], it is essential to prioritize studies on Asian American adolescents to comprehend their mental health. Although our study did not reveal significant racial/ethnic mental health disparities, we recommend further research in this area.

In this study, 24.2% of the participants reported having a family member or someone close diagnosed with COVID-19, with a significantly higher prevalence of both anxiety and PTSD. However, while the impact of depression was higher among families affected by COVID-19 compared to those not affected by COVID-19 (35% vs 22%), the significance level was marginally significant (*P*=.055), possibly due to the sample size. This finding is similar to other studies that reported having a family member or friend diagnosed with COVID-19 was significantly related to higher levels of anxiety [6].

The depression rate (25.5%) in the current study was double the pre-COVID-19 prevalence of major depressive episodes among US adolescents (13.3%) [19]. Similarly, the prevalence of anxiety (24%) was comparable to rates (24.9%) reported in China [8,9]. Our participants’ mean PTSD scores of 8.08 approached the potential cutoff scores of 10 on the IES-6 scale [17]. However, comparisons with other studies were challenging due to differences in PTSD measurement tools and limited studies during the COVID-19 pandemic among adolescents [7]. Therefore, further studies are needed to examine the prevalence of depression, anxiety, or PTSD that targets the US adolescent population, given the inconsistent findings.

As coping strategies to overcome stress during the pandemic, most participants in this study used social media and/or the internet, playing video or computer games, while fewer than half engaged in outdoor or indoor physical activities. This aligns with an Australian study where adolescents increased inactivity as well as social media and internet use during the COVID-19 pandemic were linked to reduced happiness [11]. Online connection itself has both positive and negative impacts on social relationships and mental health. However, it is evident that pandemic prevention orders—physical distancing, stay-at-home orders, and school closures—have led to increased screen time and increased use of the internet and smartphone as well as decreased physical activity among school-age children and adolescents, all of which can lead to decreased interpersonal relationship and social support. To address this, it is crucial to promote healthier coping strategies and encourage social interaction despite physical distancing. Examples of healthy coping strategies include (1) socially interactive networking to help adolescents stay connected with each other in their community [13,20,21], (2) participating in physically active indoor activities through virtual networking [13], and (3) healthy sleep management [14].

### Limitations

The use of convenience sampling, online surveys, and self-reported questionnaires may limit the generalizability of our findings and could lead to underreporting of mental health issues due to stigma. Additionally, the study is limited in its inclusion of gender-diverse and cultural factors, which may restrict the extent of perspectives captured. Nonetheless, this pioneering study offers valuable insights into the mental health of US adolescents during the 2020‐2021 COVID-19 pandemic, including a significant representation of Asian American adolescents.

### Relevance for Clinical Practice

Given the increased prevalence of depression, anxiety, and PTSD in adolescents identified in this study, it is essential to develop, implement, and expand prevention programs focusing on mental health. This includes routine screening and early detection in primary care and psychiatric/mental health care settings for both adolescents and their families. School nurses need to be aware of the impact of the COVID-19 pandemic on adolescents’ mental health. School-based programs that augment and/or complement adolescents’ use of online devices with more interactive and creative activities should be developed and implemented in collaboration with adolescents and their families. Collaboration and cooperation between health care settings, schools, families, and students should be encouraged to help identify students at risk.

### Conclusion

Approximately 25% of participants who had a family member or someone close to them diagnosed with COVID-19 were significantly associated with anxiety and PTSD. Participants in this study heavily relied on social media and the internet a lot more than any other coping strategies, and also experienced increased rates of anxiety and PTSD during the pandemic. Implementing prevention programs with routine screening, early detection, and referrals for mental health support is crucial. Additionally, school and home-based activities to enhance social interaction and healthy coping strategies among adolescents and their families should be both developed and encouraged.

## Abbreviations

GAD: generalized anxiety disorder
GAD-2: anxiety subscale
IES-6: impact of event scale
PHQ-2: depression subscale
PHQ-4: patient health questionnaire-4
PTSD: post-traumatic stress disorder
